# Radiation overdose: the utility of ct scanning in self poisoning

**DOI:** 10.1186/2197-425X-3-S1-A611

**Published:** 2015-10-01

**Authors:** G Sondh, S Noble, RI Docking, P Doherty, A Davidson, A Mackay

**Affiliations:** University of Glasgow, Medical School, Glasgow, United Kingdom; Victoria Infirmary, Glasgow, United Kingdom

## Introduction

The UK has one of the highest deliberate self-poisoning overdoses within Europe [1]. The majority of them have a short length of stay with a low mortality. Patients commonly present with a reduced GCS, triggering the order for a CT scan. This is generally despite the absence of lateralising neurology, leading to multiple scans, especially for recurrent overdoses, often providing little or no clinical benefit and at a cost of £88 per scan [2].

## Methods

This was a retrospective cohort study using data from the Scottish Intensive Care Society database and radiology archive software. Six thousand three hundred and thirty-three patients were available and 332 patients matched the parameters we set. These parameters were:

- Any ICU diagnosis that equals drug overdose/misuse
- Any ICU diagnosis that equals drug toxicity
- Any ICU diagnosis that equals self poisoning

The one hundred most recent patients within this group were then selected.

We collected demographic data, details of overdose and CT scan reports for these 100 patients using the hospital clinical information systems.

## Results

All patients admitted to the Victoria Infirmary ICU with drug overdoses between 13 January 2009 and 4 July 2014 were reviewed. One hundred patients were identified. The mean age was 38 years. Fifty-six patients were male and 44 were female.

The median (IQR) length of stay within ICU was 2 (1-3) [range (1-20)] days. Median (IQR) APACHE II score (n=82) was 17 (17-21) [range (4-40)]. Five patients (5%) died while in ICU.

Antidepressants were the most commonly abused drug with 32(18%) cases reported. The next major group was alcohol with 29(17%) cases and then benzodiazepine with 22 (13%) cases. The majority of overdoses were mixed substances. 43% of patients had CT scans taken, only 9 (20% of the 43 scanned) of these patients showed signs of head trauma and none of these cases had pathology on their CT scan. 31/34(91%) of the non-trauma cases had negative findings. The remaining 3/34 (9%) had positive CT findings and all of these significantly changed their management. Two of these patients died. Pre intubation GCS was recorded in 63% of patients. The remaining 37% had no GCS information available. The median and mode GCS were both 3.

## Discussion

Our initial hypothesis was that CT brain scanning was superfluous in the context of drug overdose and represented a defensive medical practice. The results of this audit have highlighted that very occasionally CT scanning results in discovering pathology that significantly affects subsequent management and disposal. We feel that we should continue with our current practice and that no reduction in CT scanning is indicated.

No external funding and no competing interests declared.
